# Patient-reported outcome measures obtained via E-Health tools ease the assessment burden and encourage patient participation in cancer care (PaCC Study)

**DOI:** 10.1007/s00520-021-06351-1

**Published:** 2021-06-22

**Authors:** Nicole Erickson, T. Schinkoethe, C. Eckhardt, L. Storck, A. Joos, L. Liu, P. E. Ballmer, F. Mumm, T. Fey, V. Heinemann

**Affiliations:** 1 Comprehensive Cancer Center, Ludwig-Maximilian University Clinic, Munich, Germany; 2 CANKADO Service GmbH, Cologne, Germany; 3 Hochwachtstrasse, Winterthur, Switzerland; 4 Winterthur, Switzerland; 5 Psycho-Oncology, Department of Medicine III, Ludwig-Maximilian University Clinic, Munich, Germany

**Keywords:** Nutrition, Cancer, E-health, Patient-Reported Outcome Measures (PROMs), Distress thermometer, Patient Generated Subjective Global Assessment (PG-SGA)

## Abstract

**Abstract:**

Patient-reported outcome measures obtained via E-Health tools ease the assessment burden and encourage patient participation in cancer care (PaCC Study)

**Background:**

E-health based patient-reported outcome measures (PROMs) have the potential to automate early identification of both nutrition status and distress status in cancer patients while facilitating treatment and encouraging patient participation. This cross-sectional study assessed the acceptability, accuracy, and clinical utility of PROMs collected via E-Health tools among patients undergoing treatment for stomach, colorectal, and pancreatic tumors.

**Results:**

Eight-nine percent mostly, or completely, agreed that PROMs via tablets should be integrated in routine clinical care. Men were significantly more likely to require help completing the questionnaires than women (inv.OR= 0.51, 95% CI=(0.27, 0.95), *p* = 0.035). The level of help needed increased by 3% with each 1-year increase in age (inv. OR=1.03, 95% CI=(1.01, 1.06), *p* = 0.013). On average, a patient tended to declare weight which was 0.84 kg inferior to their true weight (Bland and Altman 95 % CI=(-3.9, 5.6); SD: 2.41) and a height which was 0.95 cm superior to their true height (Bland and Altman 95 % CI=(−5, 3.1); SD 2.08). Patient-reported nutrition status was significantly associated with the professionally generated assessment (95% CI=(2.27, 4.15), *p* < 0.001). As nutrition status declined, the distress score increased (95%CI=(0.88, 1.68), *p* < 0.001). Of the patients, 48.8% who were both distressed and malnourished requested supportive care to address their problems.

**Conclusion:**

Patient-reported assessments utilizing E-health tools are an accurate and efficient method to encourage patient participation in cancer care while simultaneously ensuring that regular assessment of psycho-social and nutritional aspects of care are efficiently integrated in the daily clinical routine.

## Introduction

Medical care, including oncology care, is currently shifting from a disease-centered approach to a more personalized approach. As this shift occurs, the importance of integrating patient-reported (PR) assessments into routine care of cancer patients is increasing. Both psycho-social and nutrition interventions rely on validated screening tools designed to identify patients with a need for an intervention. However, until now, the burden of distributing, completing, and inputting data from both psycho-social and nutrition screening assessments with the purpose of triaging patient care is still placed largely on healthcare professionals (HCP) [1]. Both patients, and HCPs, can therefore benefit by harnessing the potential of E-health tools which integrate and automate assessments based on patient-reported outcomes measures (PROMs). This approach can improve efficiency, making it easier to integrate required screening and assessments into the clinical routine. Furthermore, E-health tools potentiate early identification and provide a more dynamic approach to patient-centered care by enabling and encouraging active patient participation [2, 3]. In fact, Chrischiles et al. found that among the general population, high-frequency users had higher odds of recognizing symptoms and adverse effects among users ≥ 65 years of age [4].

Therefore, the objective of this duo-centric study was to assess the acceptability, accuracy, and clinical utility of PROMs collected via E-Health tools among patients undergoing treatment for stomach, colorectal, and pancreatic tumors.

## Methods 

This cross-sectional study took place in Germany and Switzerland. The primary focus was to assess the accuracy and clinical utility of PROMs collected via E-health tools during routine care.

### Participants and data collection 

Patients diagnosed with stomach, pancreas, and colorectal cancer undergoing therapy were recruited using convenience sampling at out-patient cancer therapy centers in Germany and in Switzerland. All patients over the age of 18 who provided informed consent were eligible for inclusion in this study. Individuals with significant cognitive or functional health issues were not approached. As the goal was to assess the acceptance and accuracy of PROMs collected via E-Health tools for all patients receiving routine care, previous experience with tablet-based assessments was not considered. Exclusion criteria were limited to lack of consent and linguistic challenges as the questionnaires were provided only in German. All participants were formally asked for permission to use their de-identified data for research purposes and were given the option to opt out at any point. This study was approved by the Institutional Ethics Committee at the Ludwig Maximillian University of Munich (Reference number:19–954), Germany, and the Cantonal Ethics Committee Zurich, Switzerland (Reference number: 2018–01,129).

### E-health platform 

CANKADO’s E-health platform was utilized for all data collection. CANKADO is approved as an active Class I medical device within the European Union (registration number DE/CA59/11976/2017) and is compliant with the US Food and Drug Administration classification for Mobile Medical Devices (2015 Appendix B). CANKADO provides full patient privacy protection and data handling compliant with ICH GCP E6(R2).

### Questionnaires

After obtaining consent, patients were given a tablet asked to complete a total of 79 tablet-based questions consisting of questions pertaining to the acceptability of tablet-based questionnaires and the following 2 validated questionnaires: (1) the Patient Generated Subjective Global Assessment Short Form (PG-SGA SF) which is designed for nutrition screening and assessment and (2) the distress thermometer (DT) which is designed for psycho-social screening and assessment [5–7]. If technical problems or any difficulties arose, patients were instructed to request assistance. Upon completion of the tablet-based questions, patients returned the tablet and it was disinfected. Dietitians then completed a nutrition risk assessment using the Nutrition Risk Score (NRS-2002) and weighed and measured the patients. Same day requests for further supportive care, any difficulty completing the questionnaires, as well as any need for assistance were tracked.

### Patient Generated Subjective Global Assessment Short Form (German 18–006 v05.10.18.)

The PG-SGA SF was modified from the Subjective Global Assessment (SGA) for the oncology population by Ottery et al. It is recommended by the Oncology Nutrition Dietetic Practice Group of the Academy of Nutrition and Dietetics and has been translated and linguistically validated in many countries [8–11]. The German language validation was published in 2019, shortly before this study was initiated [12]. The PG-SGA SF comprises of 4 components: weight history, food intake, nutrition impact symptoms, and activities and function. The PG-SGA SF has demonstrated comparable sensitivity and specificity to that of the full-length scored tool [13]. Numerical scores range from 0 to 36 and a score ≥ 9 indicates a high risk for malnutrition [14].

### Nutrition Risk Score (NRS-20002)

Also, recommended by ESPEN, the NRS-2002 is a nutrition risk screening tool for hospitalized patients and is designed to be completed by the HCP. The NRS-2002 consists of three components: the severity of disease, nutritional status, and age. A score ≥ 3 indicates risk of, or existing malnutrition [15].

#### Distress thermometer (DT)

Recommended by the National Comprehensive Cancer Network (NCCN), as well as national guidelines for psycho-oncological assessment, counseling, and treatment of adult cancer patients, the DT is a psycho-social subjective test based on patient-reported (PR) data aimed at measuring the level of distress the patient is currently experiencing. It consists of a scale from 0 to 10, with 0 indicating no distress and 10 indicating extreme distress [6, 16]. While some literature recommends a cutoff score of ≥ 4 as an indication of clinically elevated distress, the German language version has been validated at a cut off score of ≥ 5 [17].

### Statistical analyses

All data was analyzed using only completers (patients without missing values). Questions related to acceptability and difficulty level of the E-health platform were formulated analogue to previous studies with each item scored on a 5-point Likert scale [18, 19]. Linear models and cumulative link models when the response distribution was discrete were used to analyze the significance of the results. When so, inverse cumulative odds ratio (inv. OR) with the corresponding 95% confidence interval (95% CI) were computed. Categorical data were presented as frequency (number) and percentage (%) and as their respective categories according to established cut off points. Proportions were compared with Chi-Squared Tests of Independence. Bland–Altman plots for weight, height, and body mass index (BMI) were used to analyze the agreement between PR and professionally measured data [20]. The Wilcoxon-Test was used to compare results between male and female patients. A linear mixed effect model using the patient as random effect and cancer type, time, and the interaction term as fixed effects was fitted to assess the weight evolution over time for each cancer type. Data analysis and presentation was done with the R system for statistical computing (version 3.6.1). All tests were two-sided and the significance level was set to 0.05.

## Results

In total,188 patients were asked to participate. Nineteen percent (*n* = 36) chose not to participate or withdrew consent. One hundred fifty-two patients (median age 62 years; range 22–86 years) completed the questionnaires. Sixty-five patients had colorectal cancer (43%); 50 patients had pancreatic cancer (33%); 25 patients had stomach cancer (16%); and the remaining 12 patients (7%) listed their diagnosis as “other.” All reported results are based on completed information only.

**Fig. 1:**
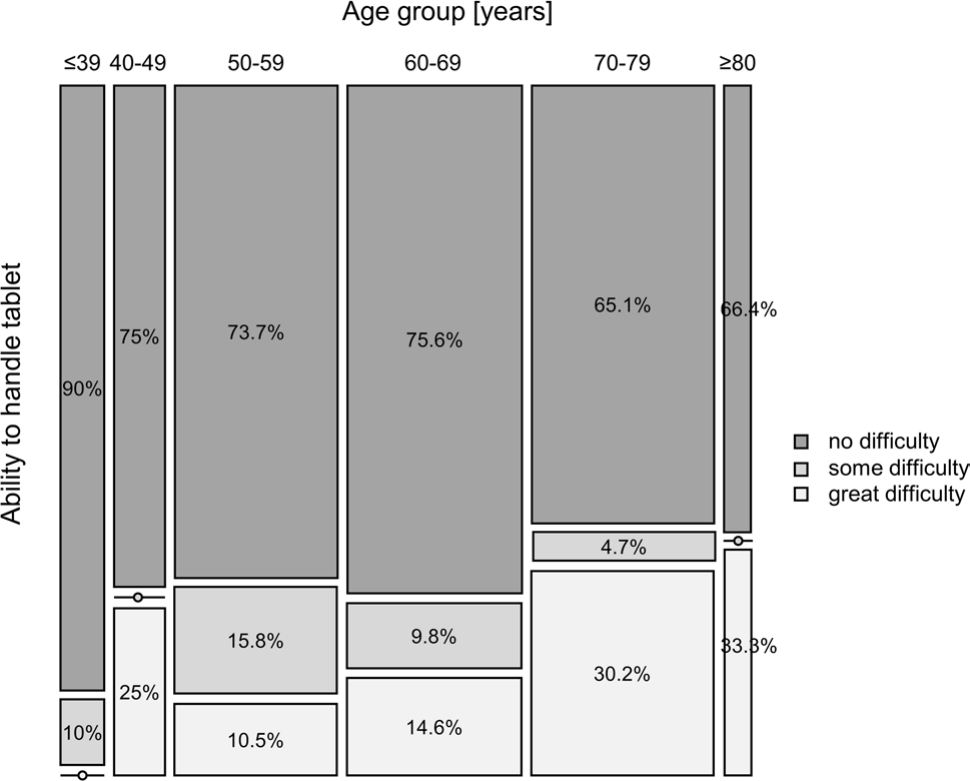
Age and ability to handle the tablet. Figure based only on completers (patients without missing values) (*n* = 152). The age group distribution was as follows: ≥ 39 years (*n* = 10); 40–40 years (*n* = 13); 50–59 years (*n* = 38); 60–69 years (*n* = 41); 70–70 years (*n* = 43); ≥ 80 years (*n* = 7)

A high proportion (89%) mostly, or completely, agreed that PROMs via tablets should be integrated in routine clinical care. While there was no evidence that gender affected this response (inv. OR = 0.62, 95% CI = (0.29,1.28), *p* = 0.207), younger patients agreed significantly more than older patients with the statement (inv. OR = 0.96, 95% CI = (0.93,0.99), *p* = 0.017). Similarly, older patients tended to find the tablet-based format more difficult to handle when compared to younger patients inv. OR = 1.03 95% CI = (1.0, 1.06), *p* = 0.052) (Fig. 1). While age was similarly distributed between the sexes, men were significantly more likely to require help completing the questionnaires than women (inv. OR = 0.51, 95% CI = (0.27, 0.95), *p* = 0.035). Additionally, the level of help needed increased by 3% with each 1-year increase in age (Inv. OR = 1.03, 95% CI = (1.01, 1.06), *p* = 0.013). Both effects remain statistically significant once adjusted for other variables.

**Fig. 2:**
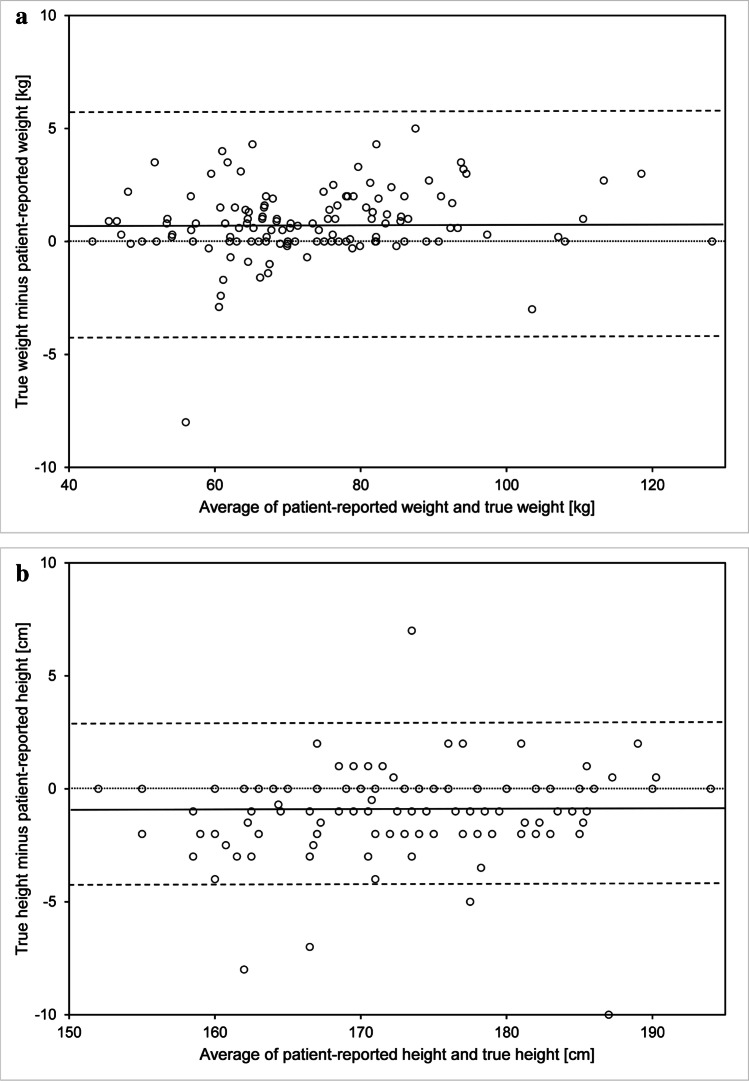
**a** Bland and Altman Plot: Patient-reported weight in kilos (kg) and true weight (kg). **b** Bland and Altman Plot: Patient-reported height in centimeters (cm) and true height (cm)

PR-height and PR-weight were closely associated with the patients’ true height and weight. In fact, 83% of the patients declared a weight which was within 2 kilos (kg) of their true weight. Likewise, 86% of patients declared a height within 2 cm (cm) of their true height. On average a patient tended to declare weight which was 0.84 kg inferior to their true weight (Bland and Altman CI = 95%; SD: 2.41) (Fig. 2a), whereas patients tended to declare a height which was on average 0.95 cm superior to their true height (Bland and Altman CI = 95%; SD 2.08) (Fig. 2b). The average BMI was 23.92 kg/m^2^, which falls within the normal range proposed by the World Health Organization. Only ten patients had a BMI below 18.5 kg/m^2^ and thus would be considered at nutrition risk based on BMI alone, while 51 patients (34%) had a BMI above the normal range.

The PR-nutrition status was significantly associated with the HCP generated assessment. In fact, a 1-unit increase in NRS-2002 was associated with an average increase in PG-SGA SF of 3.21 (95% CI = (2.27, 4.15), *p* < 0.001). These results remained significant when adjusting the data for a more normal response distribution, both when using the square root transformation and the Asinh transformation (*p* < 0.001). When the continuous scales were transformed using their respective categorical cut off scores, analogue to Zhu et a., 70% of the PR- nutrition status correlated with the HCP assessed nutrition status (Table 1) [21].

**Table 1:** Chi-Squared Tests of Independence demonstrating association between patient reported nutrition status, professionally assessed nutrition status and patient distress levels

Association between patient reported nutrition risk & professionally assessed nutrition risk (*n* = 150)						
		NRS low risk (1–2)	NRS high risk (≥ 3)			p-value
PG-SGA SF Low/medium risk ≤ 8		*n* = 56	*n* = 25			*p* < 0.001
PG-SGA SF High risk ≥ 9		*n* = 20	*N* = 49			
Association between patient reported nutrition status & patient distress levels (n = 148)						
	Distress Thermometer No ≤ 4			Distress Thermometer Yes ≥ 5	p-value	
PG-SGA SF Low/medium risk ≤ 8	*n* = 58			*n* = 23	*p* < 0.001	
PG-SGA SF High risk ≥ 9	*n* = 24			*n* = 43		
Association between professionally assessed nutrition status and distress levels (*n* = 146)						
	Distress Thermometer No ≤ 4			Distress Thermometer Yes ≥ 5	*p* value	
NRS2002 Low/medium risk ≤ 2	*n* = 51			*n* = 23	*p* < 0.001	
NRS2002 High risk ≥ 3	*n* = 30			*n* = 42		

Data reported in this table is based only on completers (patients without missing values). The number of participants therefore, reflects completers only for each category

**Fig. 3:**
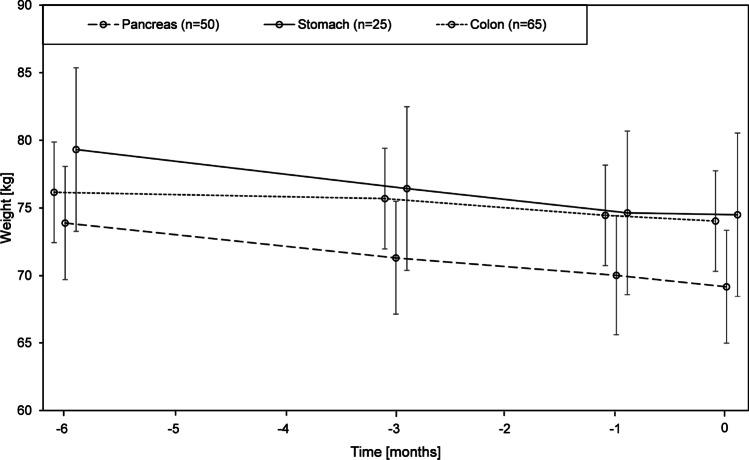
Mean patient-reported weight loss over time in kilos (kg) as compared with current patient-reported weight (kg) according to cancer type

Pancreatic and stomach cancer patients reported a mean weight loss of more than 6% (6.8 and 6.5% respectively) of their body weight when compared with their current PR-weight over the previous six months (Fig. 3). Colon cancer patients reported the least weight loss over the previous 6 months (3.9%). The PR evolution of weight over time showed a continuous weight loss trend among all three cancer types over the previous 6-month period. The average weight loss per month was 0.84 kg among stomach cancer patients (95% CI = (− 1.14, − 0.54), *p* < 0.001), followed by an average 0.78 kg loss among pancreatic cancer patients ((95% CI = (− 1.14, − 0.54), *p* < 0.001). Colon cancer patients reported a 0.5 kg loss per month ((95% CI = (− l0.68, − 0.31), *p* < 0.001).

As nutrition status declined, the distress score increased, while the activity levels declined. In fact, a 1-unit increase in PG-SGA SF score was associated with an average increase on the DT of 1.28 (95%CI = (0.88, 1.68), *p* < 0.001) and a simultaneous decrease in activity level (1 unit decrease in the activity level results in an increase on the PG-SGA-SF score of 4.75, (95% CI = (3.86, 5.64) *p* < 0.001). Similarly, there was a significant association between a rise in the PR-distress status and an increased NRS-2002 score (inv. OR = 1.33, 95%CI = (1.17, 1.52), *p* < 0.001). When the continuous scales were transformed using their respective categorical cut off scores, the significance remained (Table 1). No significant differences were found between males and females with respect to the level of distress (Wilcoxon *p* = 0.77) or the association between distress and nutrition status (*p* = 0.19).

A total of 38.2% (58/152 patients) of all patients requested supportive care in the form of nutrition interventions on the same day after completing the questionnaires. Similarly, 28.2% (43/152 patients) presented with both a declined nutrition status a distress level ≥ 5. Among these 43 patients, almost half (48.8%) requested same day supportive care. Patients who requested nutrition counseling had, on average, a 2.05 point higher PG-SGA SF score than patients who did not request counseling (95% CI = (− 0.2, 4.31), *p* = 0.074). Furthermore, patients seemed to have a good understanding of their nutrition risk. In fact, the PG-SGA score among patients who reported feeling well-nourished was, on average, 7.09 points lower than patients who did not believe that they were currently well nourished (95% CI = (4.52, 9.66), *p* < 0.001).

## Discussion

E-health tools could potentially contribute to an easy and efficient identification and treatment of patients’ care needs while simultaneously encouraging active participation on behalf of the patient [22]. However, before these integration of E-health platforms into the clinical care settings, it is important to analyze how, and if, age and gender may affect the overall acceptance of E-health applications. Similar to previous studies conducted among oncology patients regarding the collection of PROMs via E-health tools, the majority of patients (89%) agreed that such methods should be integrated into clinical care [23–26]. This result may be biased due to the fact that those patients who agreed to participate in our study and similar studies were more likely to be open to the idea of E-health tools and have less difficulty using them. However, in our study, 188 patients were asked to participate. Nineteen percent (*n* = 36) chose not to participate or withdrew consent. Only half of the patients (18/36) cited reasons specifically related to the use of E-health tools, while others cited reasons not related to E-health tools such as being too tired or not having their reading glasses on hand. Therefore, we were not able to confirm or deny this potential bias. Our results also showed a linear relationship of age to the need for assistance while utilizing our E-health application. This result confirms previous research which found that elderly people have less technological self-efficacy and a higher levels of anxiety while using innovative technology in healthcare settings and therefore may require more assistance [27]. Gender additionally affected the likelihood that patients required assistance completing the questionnaires via E-health tools with women requiring significantly less help than men. This information may be useful in identifying the best collective for future researchers planning similar interventions with E-health tools and assessing their resources available for providing assistance when required.

As PROMs become more integrated into clinical care, it is also important that their clinical accuracy and utility is reported in various populations and settings. Therefore, we analyzed the differences between PR-data regarding height and weight as well as nutrition status and compared PR-data to data obtained by HCPs via established clinical assessments methods. We further analyzed what percentage of patients requested same day supportive care after completion of the PROMs questionnaires.

While previous studies have reported biases between self-reported and measured anthropometrics, these results are influenced by settings, social aspects, as well as the method of data collection and the population being studied [28–32]. While subjects included in population studies tend to underreport their weight and overestimate their height, other population-specific studies showed that underweight participants tend to over-report their weight, whereas overweight participants tend to underreport their weight [33, 34]. In contrast, our study found that 83% of patients declared a weight within 2 kg of their true weight and a height within 2 cm of their true height. This may have been due to the fact that our patients were undergoing therapy and therefore were more inclined to be aware of their height, weight, and weight change. These results are also comparable with the NutriNet-Santé study (*n* = 2514) which concluded that the PR-anthropometric data is valid enough to be used when studying associations of nutritional factors with anthropometrics and other health outcomes [30]. It is important to note, however, that although our data revealed only few outliers, our largest outliers were 12.8 kg (underestimated) and 14.9 kg (overestimated). The patient who underestimated his/her weight by 12 kg also reported a stable weight for the previous 3 months and had a measured BMI of 28.4 kg/m^2^. Therefore, this outlier may be explained by previous studies that report the tendency of patients who are overweight to under-sreport their weight [28]. The patient who overestimated his/her weight had lost 16 kg since receiving their diagnosis, indicating that perhaps this subject was unaware of the extent of the weight loss. It is therefore important to be aware that significant weight changes, as well as patient’s perception of their BMI may influence the accuracy of PR-weight. PR outliers for height consisted of only 3 overestimations by 8, 10, and 11 cm. While studies have shown that over-reporting height of 2.5–4.5 cm is common, this only partly explains the discrepancy [28, 35]. The accuracy of PR-height in the literature is more consistent than weight. In fact, several studies show that PR-height tend to be within 2.5 cm estimate true height and can be considered an accurate and valid method for collecting this data, even among cancer patients [28, 32, 35, 36].

Height and weight make up only a small part of nutrition assessments. Thus, it is also important to assess the accuracy of other PR-nutrition assessments in comparison to HCP generated assessments. Therefore, we chose to compare the results of the NRS-2002 HCP generated nutrition assessment to that of the PR-version assessed using the PG-SGA SF. Our results were consistent with previous studies, which found significant associations between the two assessments analyzing both the continuous and categorical scales [5, 37]. In fact, other validation studies conducted among cancer patients conclude that the PG-SGA SF shows a higher specificity and sensitivity compared to various HCP based assessments like the NRS-2002 [38–40]. As the PG-SGA SF shows consistent results for accuracy and comparative, if not better identification of malnutrition, we therefore agree that it is an appropriated method to assess PR-nutrition status, as it allows quick identification and prioritization of patients and takes the assessment burden off of the HCPs.

Our PR-data revealed patients reported a significant weight loss for all three cancer types going back six months. According to international consensus criteria, weight loss > 5% over the last 6 months is classified as cancer cachexia [41]. Both pancreatic and stomach cancer patients lost on average more than 6% of their weight over a 6-month period when compared with their current weight indicating a need for optimum oncological and general medical management. It should be noted that weight loss etiology is very complex, correlated with tumor location, size and depth, and the type and length multimodal therapy provided which could not be analyzed within the framework of this study. Additionally, the patients in our study were offered regular nutrition and psychological interventions which may indicate that patients who are not offered such support could fare worse [42–44]. Scientific data relating the accuracy of weight history recall is scarce. What little data exists suggest that patients can be unclear about the magnitude of their weight change. Unfortunately, we were not able to collect HCP height and weight measurements going back in time in order to validate or compare our findings. However, as weight loss is considered to be an independent prognostic factor for decreased survival in cancer patients, more data regarding the accuracy of PR-weight recall could potentially help physicians more confidently identify patients at risk [42, 45, 46].

We were further able to track to number of study participants who requested supportive care on the same day of the study. In total, 58/152 patients (38.2%) requested supportive care in the form of nutrition therapy. The patients who requested nutrition care had PG-SGA score that was on average 2.05 points higher than patients who did not request supportive care (95% CI = (− 0.2, 4.31); *p* = 0.074). Among the patients who were both distressed and malnourished (*n* = 43), almost half (48.8%) requested supportive care. These results may indicate that integrating assessment questionnaires based on PROMs into clinical routine assessments may empower patients to actively participate in their care.

Our study had several strengths and limitations. Firstly, we were able to include centers in two different countries indicating that the results are not limited to the infrastructures, resources, and patient population at a single center. This also indicates that the E-health platform is appropriate for use in multicenter and/or international studies. As all data in this study were generated at a single-point observational basis, we could not validate the results regarding the accuracy of weight history which could be considered a limitation. While cross-sectional studies such as ours are useful for gaining insights into a population and establishing evaluation parameters and processes for future studies, our design was not as stringent as in randomized controlled trials and should thus be regarded as such. Furthermore, the inclusion criteria did not limit patients in regard to cancer stage or type of therapy and could thus not account for such potentially confounding factors. As we did not collect any data regarding social status, we cannot determine if this may have played a role in our results. The fact that we excluded individuals with linguistic challenges may have resulted in the exclusion of individuals with a migrant background and could also be considered a limitation. Additionally, patients who agreed to participate in our study were more likely to be open to the idea of E-health tools which could have created an inherent bias in the data collection. As cancer patients undergoing treatment can also experience neuropathy, finger dexterity may have played a role in their need for assistance and could have affected our results. Lastly 12 patients (7%) listed their diagnosis as “other” yet were considered eligible for the study, although the primary tumor could not be retrospectively identified we can say with confidence that it must have been one of the three types listed in our inclusion criteria.

## Conclusion

Nutrition status, distress status, symptoms, and weight history are inter-related and are all known to affect clinical outcomes. The use of PROMs obtained via E-health platforms to identify these factors could not only ease the burden of HCPs who carry out these assessments but potentially lead to early identification and treatment while simultaneously encouraging patient participation. These results support the accuracy and clinical utility of PROMs when compared to HCP generated data. Randomized controlled trials using digital platforms to obtain PROMs followed up with provision of early professional psycho-social and nutrition interventions for cancer patients at risk for malnutrition and/or distress should be conducted.

## Data Availability

The datasets generated during and/or analyzed during the current study are available from the corresponding author on reasonable request.

## Abbreviations

BMI: Body mass index
CI: Confidence interval
CM: Centimeters
DT: Distress thermometer
ESPEN: European Society for Clinical Nutrition and Metabolism
HCP: Healthcare professional
Inv. OR: Inverse odds ratio
NCCN: National Comprehensive Cancer Network
NRS-2002: Nutrition Risk Score
KG: Kilos
PG-SGA SF: Patient Generated Subjective Global Assessment Short Form
PR: Patient-reported
PROMs: Patient-reported outcome measures
QoL: Quality of life
SGA: Subjective global assessment

## Code availability

CANKADO is approved as an active Class I medical device within the European Union (registration number DE / CA59 / 11,976/2017) and is compliant with the FDA classification for Mobile Medical Devices (2015 Appendix B).
